# Differences in spine volumetric bone mineral density between grade 1 vertebral fracture and non-fractured participants in the China action on spine and hip status study

**DOI:** 10.3389/fendo.2022.1013597

**Published:** 2022-10-27

**Authors:** Yandong Liu, Aihong Yu, Kai Li, Ling Wang, Pengju Huang, Jian Geng, Yong Zhang, Yang-yang Duanmu, Glen M. Blake, Xiaoguang Cheng

**Affiliations:** ^1^Radiology Department, Peking University Fourth School of Clinical Medicine, Beijing, China; ^2^Radiology Department, Beijing Anding Hospital Capital Medical University, Beijing, China; ^3^Intervention Department, Beijing Chao-Yang Hospital, Capital Medical University, Beijing, China; ^4^South Medical Image Center, The First Affiliated Hospital of University of Science and Technology of China (USTC), Anhui, China; ^5^School of Biomedical Engineering and Imaging Sciences, King’s College London, St Thomas’ Hospital, London, United Kingdom

**Keywords:** vertebral fracture, prevalence, Genant’s semi-quantitative method, QCT, volumetric bone mineral density

## Abstract

**Purpose:**

This study evaluated the prevalence of vertebral fractures (VF) in middle-aged and elderly Chinese men and women and explored the differences in lumbar spine volumetric bone mineral density (vBMD) derived from quantitative CT (QCT) between those with a grade 1 vertebral fracture and non-fractured individuals.

**Materials and methods:**

3,457 participants were enrolled in the China Action on Spine and Hip Status (CASH) study and had upper abdominal CT examinations. Vertebral fractures were identified by Genant’s semi-quantitative method from lateral CT scout views or CT sagittal views. L1-3 vBMD was measured by Mindways QCT Pro v5.0 software. The characteristics of different fracture severity groups were compared using one-way ANOVA, independent-samples t-tests, and Kruskal-Wallis H-tests.

**Results:**

1267 males (aged 62.77 ± 9.20 years) and 2170 females (aged 61.41 ± 9.01 years) were included in the analysis. In men, the prevalence of VF increased from 14.7% at age<50 years to 23.2% at age ≥70 years, and in women from 5.1% at age<50 years to 33.0% at age ≥70 years. Differences in mean age and vBMD were found between the different fracture grade groups. After age stratification, vBMD differences in men aged < 50 years old disappeared (p = 0.162) but remained in the older age bands. There was no significant difference in mean vBMD between those with multiple mild fractures and those with a single mild fracture.

**Conclusion:**

In women, the prevalence of VF increased rapidly after age 50, while it grew more slowly in men. In general, with the exception of men <50 years old, participants with a grade 1 VF had lower vBMD than non-fractured individuals. The majority of women younger than 50 with a grade 1 VF had normal bone mass. We recommend that a vertebral height reduction ratio of <25% be diagnosed as a deformity rather than a fracture in people under the age of 50. The presence of multiple mild fractured vertebrae does not imply lower BMD.

## Introduction

Vertebral fracture (VF) is the most common osteoporotic fracture (1) but is easily missed in clinical practice because it is often asymptomatic (2). Not only can VF itself result in a poor prognosis (3), but it can also predict subsequent incident fractures (4, 5), so identification of VF, especially asymptomatic VF, is critical to prompting medical attention and preventing bad outcomes (2). Evaluation of the prevalence of VF in the population is an important aspect of public health. Cui et al. reported the prevalence of VF in postmenopausal Chinese women (6). However, the cohort of Cui’s study was limited to a single city and only included postmenopausal women over 50 years old. Until now, there has been no national data on the prevalence of VF in middle-aged Chinese women or middle-aged and elderly men. In this study, VF status in China was evaluated based on a nation-wide multi-center study (7).

The Genant semiquantitative (GSQ) method, in which vertebrae are categorized as grade 0 (non-fractured), 1 (mild), 2 (moderate) or 3 (severe) according to their reduction in height, is the most widely used criterion in epidemiological and clinical studies for evaluating osteoporotic vertebral fractures from radiographs (8–11) and was employed in the present study (8). However, the validity of Grade 1 VFs has been challenged over the years, and some researchers disregard Grade 1 deformities as a feature of VF (2, 12–15). In contrast, other research teams have presented evidence to support the relationship between Grade 1 VFs or minor vertebral deformities, bone mineral density (BMD), and further incident fractures (16–22). These studies were all based on conventional radiography or DXA-assisted vertebral fracture assessment (VFA), and bone mineral density (BMD) was evaluated by dual-energy X-ray absorptiometry (DXA) and represented by T-scores or areal BMD (aBMD, g/cm^2^). There is little literature in this field based on volumetric BMD (vBMD mg/cm^3^) derived from quantitative computed tomography (QCT), another recognized technique for diagnosing osteoporosis. Compared with DXA, a 2-dimensional method, QCT measures vBMD from a 3-dimensional image (23) and can avoid the influence caused by scoliosis, osteoarthritis of spine and calcification of vessel and/or ligament. Some studies illustrated that vBMD may be a more accurate predictor of fracture risk than aBMD (24, 25). The current study explored the differences in vBMD between participants with Grade 1 VF and those without any evidence of a vertebral radiographic deformity. Furthermore, vBMD was also compared between those with a single Grade 1 VF and those with multiple Grade 1 VFs, an aspect that, to the best of our knowledge, has not previously been discussed.

## Materials and methods

### China action on spine and hip status study

The cohort for this study was a subgroup of the China Action on Spine and Hip Status (CASH) study (NCT 01758770) (7). A total of 12 centers from 6 provinces (3 from Sichuan, 3 from Jiangsu, 1 from Shanxi, 1 from Shaanxi, 1 from Liaoning, and 1 from Jiangxi) and 1 municipality (2 from Beijing) participated in this study. The protocol and informed consent documents for the CASH study were reviewed and approved by the institutional review board of the Beijing Jishuitan Hospital (approval numbers No. 201210-01; No. 201512-02). The inclusion criteria are that participants should be aged over 40 years old and able to give informed consent. Exclusion criteria are pregnant women, individuals with metal implants in the lumbar spine, use of medications or the existence of any disease or condition known to have a major influence on BMD, and inability to give informed consent.

### Participants and data collection

The CASH study CT scans were performed between March 2013 and August 2017. All participants lived near one of the 12 centers and were willing to undergo a spine CT scan. A total of 3457 participants between 40 and 82 years old were enrolled in the study.

For most participants, social-demographic data, height, weight, waist circumference (WC), and hip circumference (HC) were recorded by a trained health physician before or after their CT scan. For the others, the information was supplemented by the baseline data based on the assumption that those data did not change in the follow-up period. Body mass index (BMI) and waist-hip ratio (WHR) were calculated by weight (kg)/height squared (m2) and WC (cm)/HC (cm) respectively.

### Quantitative computed tomography volumetric bone mineral density measurement

Mindways (Austin, TX, USA) QCT phantom and software were used at all centers. The phantom was scanned with each participant to ensure the accuracy and precision of the vBMD measurements. Participants lay on the phantom and had upper abdominal CT examinations with a fixed table height and scan parameters. At the same time, the CT scout views including the T4-S1 vertebrae were obtained. The detailed scan protocol was reported in a previous paper (7). To eliminate any discrepancy between different CT scanners, ten scans of a European spine phantom (ESP, No.145) were performed on each CT scanner. All QCT data were transferred to the Beijing Jishuitan Hospital for analysis and quality control.

The L1 to L3 vertebrae vBMD values were measured by Mindways QCT Pro v5.0 software according to the manufacturer’s protocol. For each participant, the average value of L1 to L3 vBMD was calculated and obtained through cross-calibration. The final value was regarded as the lumbar spine vBMD value. Following the criteria of the International Society for Clinical Densitometry (ISCD) 2007 (26) and the Chinese Expert Consensus on the Diagnosis of Osteoporosis (27), a vBMD value ≥120 mg/cm^3^ was defined as normal, a value between 80 and 119 mg/cm^3^ as osteopenia, and a value < 80mg/cm^3^ as osteoporosis.

### Identification of vertebral fracture

The lateral CT scout view images of 3340 participants were evaluated by an expert MSK radiologist (XGC) with many years of experience in vertebral fracture assessment. The digital images were displayed and viewed with a professional DICOM view workstation. The capability and reliability of lateral CT scout views in assessing vertebral fractures has been verified (28, 29). Under ideal conditions, CT scout view images can assess the T4-L4 vertebrae. However, due to limitations in actual scanned area or overlapping of ribs, all T4-L4 vertebrae could be assessed in only 683 images and all T5-L4 vertebrae in 864 images. In most cases, the assessable range was from T6 to L4. The fracture status of another 117 participants was diagnosed based on the CT sagittal views of the T10-L4 vertebrae because of the lack of CT scout views.

**Figure 1:**
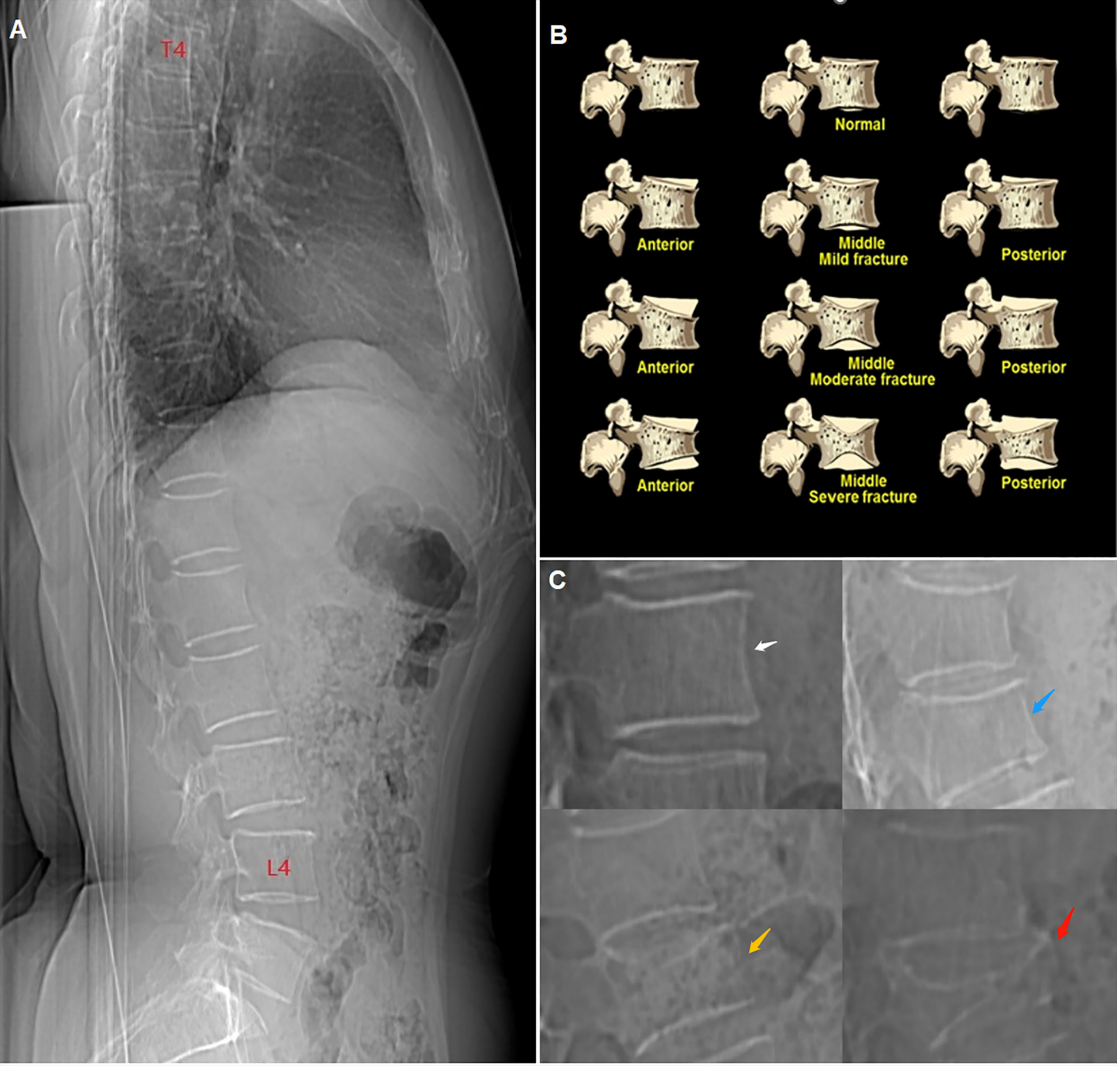
**(A)** CT lateral scout view of the vertebrae from T4 to L4. **(B)** schematic diagram of Genant’s semiquantitative (GSQ) method. **(C)** compression degree of vertebrae showed in CT lateral scout view, white arrow: non-fractured (grade 0), blue arrow: mild (grade 1), yellow arrow: moderate (grade 2), red arrow: severe (grade 3).

Genant’s semi-quantitative (GSQ) method (8) was used as the criterion of vertebral fracture: vertebral height reductions of>20% to 25%,>25% to 40%, and>40% were defined as grade 1 (mild), grade 2 (moderate), and grade 3 (severe), respectively (**Figure 1**). Vertebrae with a grade ≥1 were identified as fractured, and the fracture severity of each individual was decided by the highest grade in that person. Grade 2 and grade 3 were merged into a single group for further analysis. Finally, all cases were divided into three groups: a non-fractured group; a grade 1 group; and a grade 2 and 3 group. The grade 1 group was further divided into two sub-groups according to the number of fractured vertebra (FV): FV=1 and FV≥2.

### Statistical analyses

vBMD was the observed variable in this study, while age, BMI, and WHR were the potential covariates. All the results were gender-specific. A comparison of vBMD, age, BMI, and WHR between the non-fracture group, the grade 1 group, and the grade 2 and 3 group was performed first. Then, the non-fracture group was compared with the grade 1 group after age stratification. Characteristics of sub-groups by the number of fractured vertebrae in the grade 1 group in different age bands were also compared. Continuous variables were shown as the mean ± standard deviation (SD), and ordinal categorical variables as numbers (n) and percentages (%). For the comparison of multiple sets of continuous variables, one-way ANOVA followed by a Bonferroni *post hoc* test was used if the variances were equal. Otherwise, Tamhane’s T2 test was chosen. Two groups of continuous variables were tested by an independent-samples t-test. Ordinal and categorical variables were tested with a Kruskal-Wallis H-test. Covariance analysis was used to eliminate the influence of covariates. Statistical analyses were performed by IBM SPSS Statistical 26.0 software. P<0.05 was considered statistically significant.

## Results

Twenty of 3457 participants were excluded. For six participants, their age or sex did not match the CASH database. Another 14 participants were missing their vBMD results. The statistical analysis included a total of 3437 participants, among whom there were 1267 males aged 62.77 ± 9.20 years and 2170 females aged 61.41 ± 9.01 years.

### Prevalence

All participants were grouped into four age bands for men and the same number for women; those aged<50 years, 50-59 years, 60-69 years, and ≥70 years, respectively. The prevalence of VF and osteoporosis in men and women, respectively, is shown in **Table 1**, together with the corresponding average vBMD results. In men, the prevalence of VF increased from 14.7% at age<50 years to 23.2% at age ≥70 years, with the percentage of osteoporotic men increasing from 3.1% to 36.5%, while the average vBMD decreased from 139.90 ± 31.61mg/cm^3^ to 92.63 ± 33.61 mg/cm^3^. In women, the prevalence of VF increased from 5.1% at age<50 years to 33.0% at age ≥70 years, with the percentage of osteoporotic women increasing from 1.6% to 69.3%, while the average vBMD decreased from 151.04 ± 34.06 mg/cm3 to 68.47 ± 31.47 mg/cm3.

**Table 1:** The prevalence of vertebral fractures (VFs), osteoporosis (OP) and the mean ± standard deviation of vBMD by gender and age bands.

Age		<50y	50-59y	60-69y	≥70y	Total
Sex						
Male	Total Number (n)	129	293	522	323	1267
	VF Number(n) and Prevalence (%)	19 (14.7)	48 (16.4)	94 (18.0)	75 (23.2)	236 (18.6)
	OP Number(n) and Prevalence (%)	4 (3.1)	22 (7.5)	80 (15.3)	118 (36.5)	224 (17.7)
	vBMD (mg/cm^3^)	139.90 ± 31.61	120.89 ± 29.07	110.22 ± 32.87	92.63 ± 33.61	111.23 ± 34.96
Female	Total Number (n)	254	604	882	430	2170
	VF Number(n) and Prevalence (%)	13 (5.1)	41 (6.8)	140 (15.9)	142 (33.0)	336 (15.5)
	OP Number(n) and Prevalence (%)	4 (1.6)	77 (12.7)	373 (42.3)	298 (69.3)	752 (34.7)
	vBMD (mg/cm^3^)	151.04 ± 34.06	117.78 ± 34.63	87.24 ± 29.51	68.47 ± 31.47	99.49 ± 40.91

VF, vertebral fracture; OP, osteoporosis; vBMD, QCT volumetric bone mineral density; y, years.

**Figure 2:**
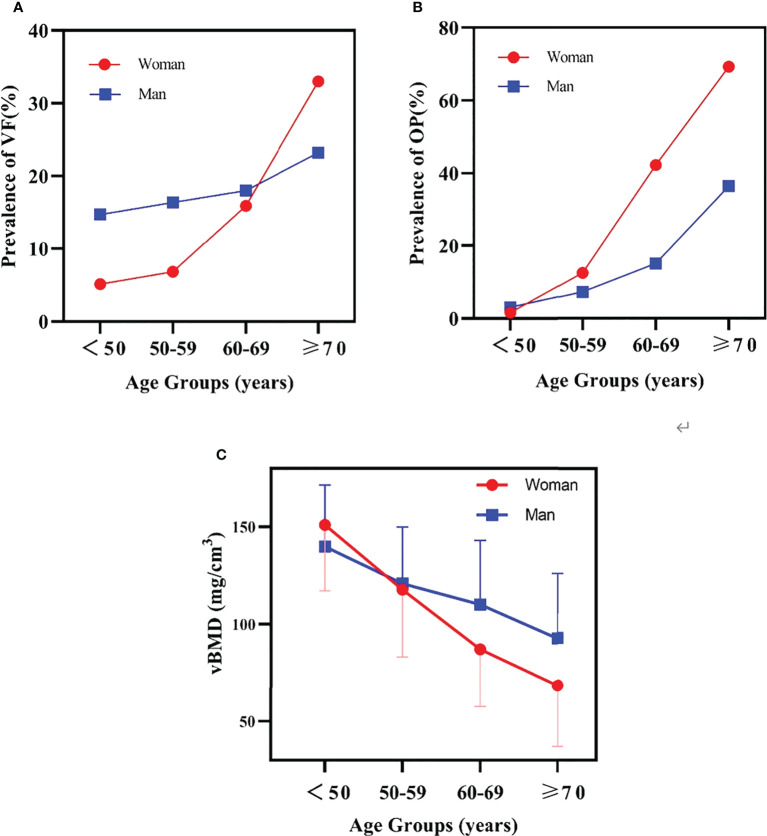
**(A)** the prevalence of vertebral fracture (VF) variation with age. **(B)** the prevalence of osteoporosis (OP) variation with age. **(C)** the mean and SD of bone mineral density (BMD) variation with age.

**Figure 3:**
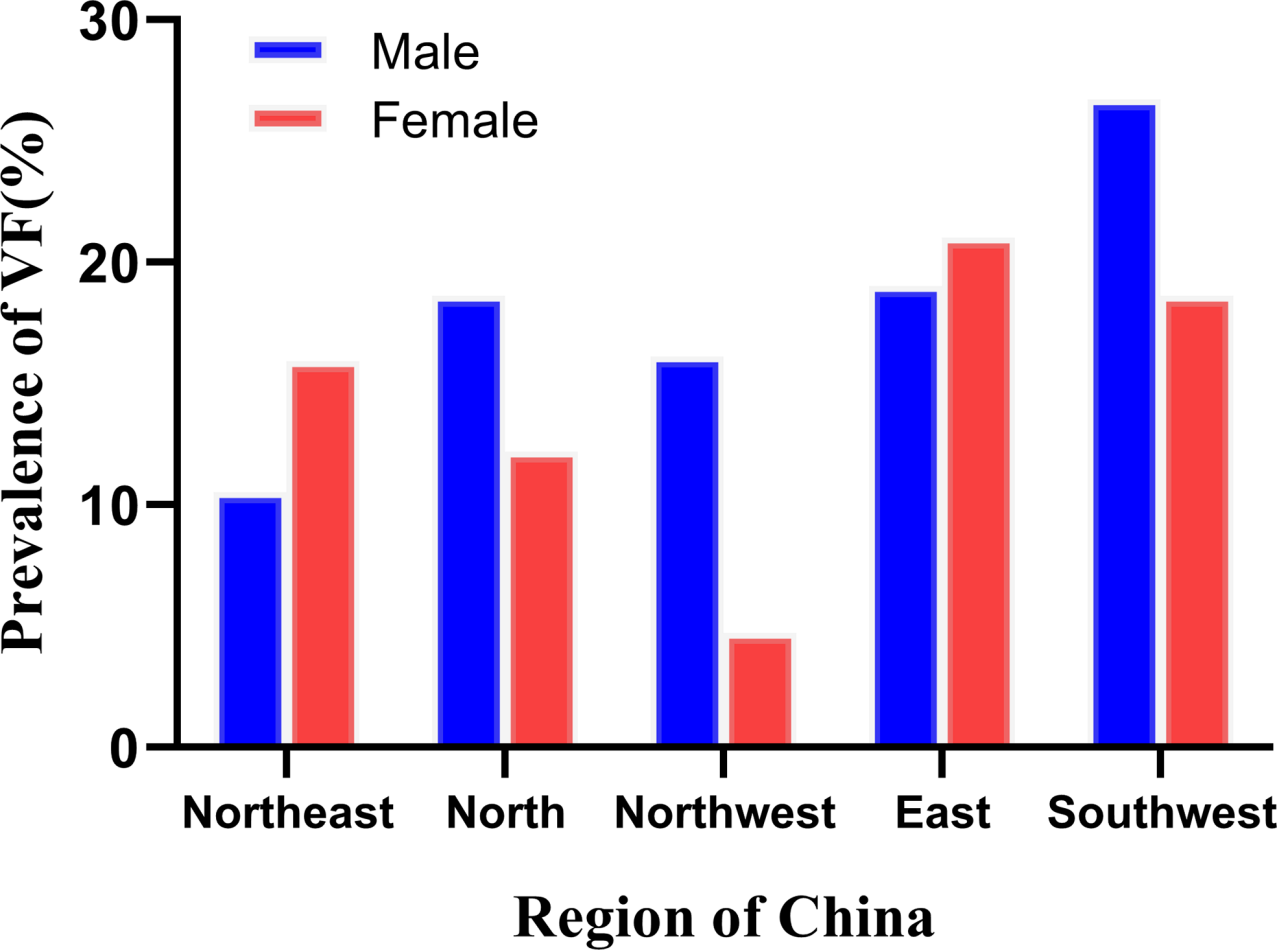
The prevalence of vertebral fracture (VF) in the ≥50 years group across different regions of China.

**Figure 2** shows plots of the prevalence of VF and osteoporosis and the variation of vBMD with age. The male prevalence of VF was much higher than the female prevalence in the group less than 50 years old, while it was lower in the group ≥70 years old. The prevalence crossed over for the group aged 60 to 69 years (women 15.9%, men 18.0%). Compared with VF, the osteoporosis prevalence cross-over point is earlier: between 50 and 59 years. In those aged<50 years, the percentage of osteoporotic women was lower than in men, and the average vBMD was higher accordingly. At 50-59 years, female vBMD (117.78 ± 34.63 mg/cm^3^) was slightly lower than male (120.89 ± 29.07 mg/cm^3^) and the prevalence of osteoporosis was slightly higher (women 12.7%, men 7.5%). The difference increased with increasing age. At age ≥70, the prevalence of osteoporosis in women (69.3%) was nearly twice that in men (36.5%). The prevalence of VF in the group ≥50 years old among the different geographic regions of China is shown in **Figure 3**.

### Characteristics

The characteristics of participants by gender and vertebral fracture grades identified by Genant’s semi-quantitative criteria are shown in **Table 2**. Male age did not differ significantly between the grade 1 group (63.57 ± 9.3 years) and the other two groups, but the age of the non-fracture group (62.47 ± 9.16 years) differed significantly from the grade 2 and 3 group (67.03 ± 9.04 years) with p = 0.010. The non-fracture, grade 1, and grade 2 and 3 groups had vBMD values of 114.47 ± 34.88 mg/cm^3^, 99.24 ± 30.71 mg/cm^3^, and 84.99 ± 34.74 mg/cm^3^ respectively. The differences between the non-fractured and the two fracture groups were statistically significant, while the difference between the two fracture groups was non-significant. In the non-fracture group, the proportion of people with normal bone density is the largest (43.9%), while the proportion with osteoporosis is the smallest (15.4%). Most men in the grade 1 group (47.0%) and grade 2 and 3 group (47.2%) had osteopenia, while the latter group had the highest proportion with osteoporosis (36.1%). There were no significant differences among the three groups in BMI (p=0.649) or WHR (p=0.824).

**Table 2:** Characteristics of eligible participants by gender and vertebra fracture grades identified by Genant’s semi-quantitative criteria.

Characteristics	Male				Female			
	Non-fracture(n=1031)	Grade 1(n=200)	Grade 2and3(n=36)	p	Non-fracture(n=1834)	Grade 1(n=237)	Grade 2and3(n=99)	p
Age(y)	62.47 ± 9.16	63.57 ± 9.3	67.03 ± 9.04	0.006^▲^	60.37 ± 8.82	65.98 ± 8.06	69.77 ± 6.67	<0.001^△^
vBMD (mg/cm^3^), n (%)	114.47 ± 34.88	99.24 ± 30.71	84.99 ± 34.74	<0.001^*^^▲^^▲^	105.31 ± 39.63	75.00 ± 32.62	50.25 ± 23.82	<0.001^*^△^^
Normal	453 (43.9)	53 (26.5)	6 (16.7)	<0.001	601 (32.8)	21 (8.9)	1 (1.0)	<0.001
Osteopenia	420 (40.7)	94 (47.0)	17 (47.2)		713 (38.9)	75 (31.6)	7 (7.0)	
Osteoporosis	158 (15.4)	53 (26.5)	13 (36.1)		520 (28.3)	141 (59.5)	91 (92.0)	
BMI (kg/m^2^)	24.19 ± 3.31	24.03 ± 3.27	23.77 ± 2.87	0.649	24.28 ± 3.49	24.59 ± 3.93	24.38 ± 3.05	0.441
WHR	0.88 ± 0.07	0.88 ± 0.07	0.88 ± 0.11	0.824	0.83 ± 0.07	0.85 ± 0.07	0.86 ± 0.07	<0.001^▲▲▲^

y, years; p, p-value; vBMD, QCT volumetric bone mineral density; BMI, body mass index; WHR, waist-to-hip ratio.

Continuous variables were shown as mean ± standard deviance, and were tested by ANOVA; ordinal categorical variables were shown as frequency(n) and percentage (%), and were tested by Kruskal-Wallis H tests.

^*^, the p -value is always<0.001 before and after being adjusted by age.

^▲^, male age shows no significant difference between Grade 1 group and other two groups, while age of non-fracture group is significantly different from Grade 2and3 group with p=0.010.

^△^,the p-value of all comparison among groups <0.001.

^▲▲^,the p-value among Grade 1 group and Grade 2and3 group =0.065, while p-value of other pairs<0.001.

^▲▲▲^, the p-value among Grade 1 group and Grade 2and3 group =1.000, while p-value of non-fracture group vs. Grade 1 group<0.001 and p-value of non-fracture group vs. Grade 2and3 group=0.002.

In women, the average age of grade 2 and 3 vertebral fracture cases (69.77 ± 6.67 years) was significantly older than that of grade 1 cases (65.98 ± 8.06 years), and the latter was significantly older than the non-fracture participants (60.37 ± 8.82 years). Grade2 and 3 cases showed the lowest vBMD value (50.25 ± 23.82 mg/cm^3^) and the highest percentage with osteoporosis (92.0%), while the non-fracture participants had the highest vBMD (105.31 ± 39.63 mg/cm^3^) and the lowest percentage with osteoporosis (28.3%). The grade 1 group was located between the other two. After controlling for age, the differences between groups remained significant (p<0.001). There was no significant difference among the three groups in BMI (p=0.441). The WHR of the non-fracture group (0.83 ± 0.07) was significantly different from the grade 1 (0.85 ± 0.07) and grade 2 and 3 groups (0.86 ± 0.07), while the latter two were the same (p=1.000). However, the difference between the non-fracture group and fracture groups became non-significant after stratification by age.

### Comparison of volumetric bone mineral density after age-stratification

In this part of the study, male and female participants were divided into three age bands; those aged<50 years, 50-65 years, and ≥ 65 years, respectively. A comparison of age and vBMD between the non-fracture group and the grade 1 vertebral fracture group is shown in **Table 3**. There was no significant difference between the two groups in mean male age after stratifying by age. In men, the mean BMD of the grade 1 vertebral fracture group was lower than the non-fracture group in all three age bands, but there was no statistical difference for those<50 years old (p=0.162). In contrast, the differences were statistically significant in the other two age bands. Participants with normal bone density in the age band<50 years accounted for 70.6% of the grade 1 group compared with 80.0% of the non-fracture group, a difference that was not statistically significant. The percentage of participants with normal bone density in the grade 1 group decreased to 29.3% and 16.8% in the 50-65 year and ≥65 year age bands respectively. Meanwhile, the prevalence of osteoporosis increased to 15.8% and 39.6% respectively. These proportions were significantly different from the non-fracture group.

**Table 3:** Comparison of age and vBMD by gender between non-fracture group and Grade 1 vertebral fracture (VF) group in different age bands. .

Agebands	Characteristics	Male			Female		
		Non-fracture	Grade 1	p_1_	Non-fracture	Grade 1	p_1_
<50y	Number	110	17	–	241	12	–
	Age (y)	46.00 ± 2.09	46.18 ± 2.40	0.751	46.07 ± 2.30	47.00 ± 1.41	0.050
	vBMD (mg/cm^3^), n (%)	141.80 ± 32.6	130.23 ± 23.46	0.162	152.41 ± 32.63	128.52 ± 47.84	0.016
	Normal	88 (80.0)	12 (70.6)	0.341	201 (83.4)	7 (58.3)	0.034
	Osteopenia	18 (16.4)	5 (29.4)		37 (15.4)	4 (33.3)	
	Osteoporosis	4 (3.6)	0 (0)		3 (1.2)	1 (8.4)	
50-64y	Number	468	82	–	950	83	–
	Age (y)	58.34 ± 4.36	57.91 ± 4.51	0.548	57.64 ± 4.31	59.72 ± 3.85	<0.001
	vBMD (mg/cm^3^), n (%)	121.46 ± 31.69	104.85 ± 25.57	<0.001	109.45 ± 34.48	90.16 ± 28.66	<0.001^*^
	Normal	233 (49.7)	24 (29.3)	0.002	334 (35.1)	10 (12.1)	<0.001
	Osteopenia	195 (41.7)	45 (54.9)		432 (45.5)	45 (54.2)	
	Osteoporosis	40 (8.6)	13 (15.8)		184 (19.4)	28 (33.7)	
≥65y	Number	453	101	–	643	142	–
	Age (y)	70.73 ± 4.28	71.08 ± 4.40	0.459	69.77 ± 4.04	71.25 ± 4.27	<0.001
	vBMD (mg/cm^3^), n (%)	100.60 ± 32.45	89.48 ± 31.21	0.002	81.54 ± 30.07	61.62 ± 23.78	<0.001^*^
	Normal	132 (29.1)	17 (16.8)	0.004	66 (10.3)	4 (2.8)	<0.001
	Osteopenia	207 (45.7)	44 (43.6)		244 (37.9)	26 (18.3)	
	Osteoporosis	114 (25.2)	40 (39.6)		333 (51.8)	112 (78.9)	

VF, vertebral fracture; VF, fractured vertebra; vBMD, QCT volumetric bone mineral density; BMI, body mass index; WHR, waist-to-hip ratio; y, years; p, p-value.

Continuous variables were shown as mean ± standard deviance, and were tested by independent-samples t-test; ordinal categorical variables were shown as frequency(n) and percentage (%), and were tested by Kruskal-Wallis H tests.

^*^, the p -value is always<0.001 before and after being adjusted by age.

For women, age was significantly different between the non-fracture and grade 1 vertebral fracture groups in the 50-65 year and ≥65 year age bands (p<0.001), but not at age<50 years (p = 0.050). In the non-fracture group, vBMD in females was always significantly higher than in the grade 1 vertebral fracture group. These differences remained after adjustment for age. In the grade 1 group, 58.3% of participants in the<50 year age band had normal bone density. For ages between 50-65 years, most female participants had osteopenia (54.2%), and the percentage with osteoporosis (33.7%) was higher than those with normal bone density (12.1%). Of the women aged ≥65 years in the grade 1 group, 78.9% were osteoporotic. The proportion was statistically significantly higher than the non-fracture group for all age bands.

The grade 1 vertebral fracture group was divided into two sub-groups according to the number of fractured vertebrae (FV): single fracture (FV=1) and multiple fractures (FV≥2) (**Table 4**). There were no significant differences between any pairs of results in **Table 4**.

**Table 4:** Comparison of age and vBMD in the Grade 1 vertebral fracture (VF) group by number of fractured vertebra (FV) at different age bands^♦^.

Agebands	Characteristics	Male			Female		
		FV=1	FVs≥2	p	FV=1	FVs≥2	p
50-64y	Number	48	34	–	73	10	–
	Age (y)	58.25 ± 4.20	57.44 ± 5.00	0.427	60.00 ± 3.64	57.7 ± 4.86	0.076
	vBMD (mg/cm^3^), n (%)	102.80 ± 21.32	107.74 ± 30.79	0.392	91.30 ± 27.32	81.80 ± 37.32	0.329
	Normal	12 (25)	12 (35.3)	0.604	9 (12.3)	1 (10.0)	0.512
	Osteopenia	28 (48.3)	17 (50.0)		41 (56.2)	4 (40.0)	
	Osteoporosis	8 (17.7)	5 (14.7)		23 (31.5)	5 (50.0)	
≥65y	Number	64	37	–	106	36	–
	Age (y)	70.95 ± 4.47	71.30 ± 4.39	0.707	71.40 ± 4.30	70.81 ± 4.20	0.475
	vBMD (mg/cm^3^), n (%)	92.70 ± 30.85	83.90 ± 31.45	0.173	61.96 ± 24.43	60.63 ± 22.08	0.773
	Normal	12 (18.7)	5 (13.5)	0.857	4 (3.7)	0 (0)	0.463
	Osteopenia	30 (46.9)	14 (37.8)		20 (18.9)	6 (16.7)	
	Osteoporosis	22 (34.4)	18 (48.7)		82 (77.4)	30 (83.3)	

VF, vertebral fracture; FV, fractured vertebra; vBMD, QCT volumetric bone mineral density; y, years; p, p-value.

^♦^, <50y group is excluded due to small sample size.

Continuous variables were shown as mean ± standard deviance, and were tested by independent-samples t-test; ordinal categorical variables were shown as frequency(n) and percentage (%), and were tested by Kruskal-Wallis H tests.

## Discussion

In this nation-wide multi-center study of 3457 Chinese middle-aged and elderly adults, we evaluated the prevalence of VFs by identifying fractured vertebra from 3340 lateral CT scout views and 117 CT sagittal views and used volumetric BMD derived from QCT to calculate the prevalence of osteoporosis. Volumetric BMD was compared between the Grade 1 VF population and the non-fractured population, as well as between subgroups of the Grade 1 VF population according to the number of fractured vertebrae.

The results show that the prevalence of VFs and osteoporosis increases with age in both men and women. Men before the age of 60 are approximately three times more likely than women to experience VFs, but this is reversed after the age of 70 when the prevalence of VF in females is approximately 1.5 times that of males (33.0% versus 23.2%), with the cross-over occurring in the 60-69 age band. In comparison, the cross-over in the prevalence of osteoporosis occurs earlier at around age 50. Women between the ages of 50 and 59 have similar vBMD to men, but at younger ages vBMD is higher in women than men. This observation is consistent with the cross-sectional study of 69,095 Chinese adults published by Cheng et al. (30). However, the prevalence of osteoporosis in the present study population aged 50 years and older is higher than that reported by Cheng et al., with the percentages of women and men over 50 with osteoporosis reaching 38.9% and 29.3%, respectively, while in the study of Cheng et al. those numbers were 29.0% and 13.5%, respectively (30). This might be due to a different age distribution between the two populations. In the present study, the percentage of people aged ≥65 years is 38.25%, but there was only 13.26% of people over 65 years in the study of Cheng et al. (30). However, after age-stratification the prevalence of osteoporosis is similar in the two studies.

Cui et al. evaluated the prevalence of VFs in postmenopausal women in Beijing, China, based on conventional radiographs and the GSQ method. The percentages were 13.4%, 22.6%, 31.4%, and 58.1%, respectively, for women aged 50-59 years, 60-69 years, 70-79 years, and ≥80 years (6). The first two numbers are higher than the corresponding results reported in the present paper (6.8% and 15.9% respectively). The discrepancy might be due to the use of different types of radiographic images in the two studies, to genuine differences between the two study populations, or to inter-observer differences. However, our results are similar to those reported by Xu et al. (31), which were based on conventional radiographs and a morphometry method developed by Black et al. (32). A multi-center study in America reported a total prevalence of 3.2% of GSQ VF in middle-aged women of different races, with a prevalence of 3.4% in Chinese American women (33). To our knowledge, the prevalence of VF in Chinese men has not been reported before and is seldom discussed in other countries. A study in a Spanish cohort reported that 21.3% of Spanish men over 50 years old suffered from VFs identified by the GSQ method (34). Our percentage is slightly higher than the present results (19.06%). This study is the first to report the prevalence of VF in people ≥50 years old across different regions of China. Men from the Southwest and women from the East had the highest VF prevalence, and men from the Northeast and women from the Northwest had the lowest VF prevalence. However, the sample size from the Northwest was small (86 women and 62 men aged ≥50 years), which limits the statistical reliability of the results.

Before age-stratification, there was a significant difference in vBMD values in men between the non-fracture and the grade 1 groups. However, this difference was not seen in men aged<50 years old, which implies that those with a vertebral height reduction of<25% were more likely to have deformities caused by degeneration or other disease rather than fractures associated with decreased vBMD. In women aged<50 years old, although mean vBMD of the grade 1 group is significantly lower than the non-fracture group, more than half of women in the grade 1 group had normal bone density. This suggests that in women too, the grade 1 group might include a significant proportion of individuals with vertebral deformities. Hence, unless there is a decrease in bone density measured by DXA or QCT, or adequate radiographic evidence of a fracture such as distortion of an endplate and/or cortex (11), we prefer to diagnose a vertebral height reduction ratio<25% in those aged<50 years old as a deformity rather than a fracture, especially in men. For individuals aged ≥50 years old, the grade 1 group has lower mean lumbar spine vBMD values compared with the non-fractured group in both men and women. This is consistent with previous studies. Lentle et al. compared DXA derived aBMD in the L1-4, femoral neck, and total hip sites between the VF and non-fractured groups and demonstrated lower aBMD values in the GSQ grade 1 VF group in both men and women aged ≥50 years (22). Johansson et al. found that older Swedish women aged from 75 to 80 years with grade 1 VFs diagnosed by VFA had lower DXA derived aBMD than those without VF in both the lumbar spine and femoral neck (19). Most studies have tended to focus on the relationship between fracture severity and BMD, but less on the relationship between the number of fractured vertebral bodies and BMD. The present study examined this issue and found no correlation between the two variables for grade 1 fractures. In other words, the presence of multiple mild fractured vertebrae does not of itself imply lower BMD.

There are several strengths in the present study. It is a nation-wide multi-center study with participants from 6 geographic regions across China. QCT derived volumetric BMD is superior to DXA derived areal BMD in avoiding the overestimation of values caused by spinal degenerative changes (35, 36). There are also limitations to this study. Due to limitations in the scanned area or overlapping of ribs, not all T4-L6 vertebrae were evaluated, and therefore we might have underestimated the prevalence of VF. Because of limited access, the study did not include hip BMD. Finally, this is a cross-sectional study that was unable to predict future incident fractures.

In conclusion, we examined the prevalence of vertebral fractures in China by age and gender. In women, the prevalence of VF increased rapidly after age 50 along with a rapid decrease in vBMD, while in men it grew more slowly along with a relatively gradual decrease in vBMD. Volumetric BMD of participants with grade 1 vertebral fracture and non-fractured individuals were compared for different age ranges. In general, with the exception of men <50 years old, participants with grade 1 vertebral fracture had lower vBMD than non-fractured individuals. The majority of women younger than 50 years old with a grade 1 vertebral fracture had normal bone mass. We recommend diagnosing a vertebral height reduction ratio of < 25% as a deformity rather than a fracture in people under the age of 50. The presence of multiple mild fractured vertebrae does not of itself imply lower BMD.

## Data availability statement

The original contributions presented in the study are included in the article/supplementary material. Further inquiries can be directed to the corresponding author.

## Ethics statement

The studies involving human participants were reviewed and approved by the institutional review board of the Beijing Jishuitan Hospital (approval numbers No. 201210-01; No. 201512-02). The patients/participants provided their written informed consent to participate in this study.

## Author contributions

YL and XC: Designed the study and conceived the report. YL: Wrote the draft of the manuscript. GB: Edited the manuscript. AY and LW: Funding acquisition and revised the draft critically. KL and JG: Data acquisition and processing. XC: Evaluated vertebral fracture. YL and PH: Statistical analysis, and created the figures and tables. CASH study team: CT scanning. YZ and Y-YD: conducted the cross-calibration CT scans. All authors contributed to the article and approved the submitted version.

## Funding

This work is supported by the National Natural Science Foundation of China (No.81971617), Beijing Hospitals Authority Youth Programme (QML20200402), and Beijing Hospitals Authority Clinical Medicine Development of Special Funding Support (ZYLX202107).

## Conflict of interest

The authors declare that the research was conducted in the absence of any commercial or financial relationships that could be construed as a potential conflict of interest.

## Publisher’s note

All claims expressed in this article are solely those of the authors and do not necessarily represent those of their affiliated organizations, or those of the publisher, the editors and the reviewers. Any product that may be evaluated in this article, or claim that may be made by its manufacturer, is not guaranteed or endorsed by the publisher.

## Abbreviations

VF, vertebral fracture; GSQ, Genant semiquantitative; BMD, bone mineral density; DXA, dual-energy X-ray absorptiometry; aBMD, areal bone mineral density; vBMD, volumetric bone mineral density; VFA, vertebral fracture assessment; QCT, quantitative computed tomography; CASH, China Action on Spine and Hip Status; WC, waist circumference; HC, hip circumference; BMI, body mass index; ESP, European spine phantom; ISCD, International Society for Clinical Densitometry; FV, fractured vertebra; SD, standard deviation.
